# Metagenome-assembled genomes from mineral tundra soils in Rásttigáisá, northern Norway

**DOI:** 10.1099/acmi.0.000655.v3

**Published:** 2024-01-23

**Authors:** Igor S. Pessi, Aino Rutanen, Jenni Hultman

**Affiliations:** ^1^​ University of Helsinki, Helsinki, Finland; ^2^​ Helsinki Institute of Sustainability Science (HELSUS), Helsinki, Finland; ^3^​ Finnish Environment Institute (Syke), Helsinki, Finland; ^4^​ Natural Resources Institute Finland (Luke), Helsinki, Finland

**Keywords:** *Candidatus* Dormibacterota, metagenomics, tundra soils

## Abstract

Microbial communities in tundra soils remain largely unknown despite their important roles in the cycling of greenhouse gases. Here, we report 59 non-redundant metagenome-assembled genomes (MAGs) recovered from mineral tundra soils in Rásttigáisá, northern Norway. The MAGs were obtained by clustering contigs according to tetranucleotide frequency and differential coverage and were manually curated to remove contigs with outlying GC content and/or mean coverage. Most MAGs were assigned to the bacterial phyla *Candidatus* Dormibacterota (*n*=12), Verrucomicrobiota (*n*=10), and Acidobacteriota (*n*=9). All archaeal MAGs (*n*=4) belong to the genus *Candidatus* Nitrosopolaris (phylum Thermoproteota). The 59 Rásttigáisá MAGs expand our knowledge of the diversity and ecological roles of tundra microbiomes.

## Data Summary

The data generated in this study have been submitted to the European Nucleotide Archive (ENA) under the project PRJEB49283. Raw reads are available under accessions ERR11584940–ERR11584949, and accession numbers for the assembled genomes are listed in Table S1, available in the online version of this article.

## Introduction

Human activities such as unsustainable industrial and agricultural practices are driving irreversible changes in tundra soils and other polar ecosystems. Greenhouse gases (GHGs) play a major role in climate change and, if emissions are not curbed drastically, atmospheric temperatures in the Arctic may be 7 °C warmer by the end of the century [1]. Microbial communities in the tundra play key roles in both the production and consumption of greenhouse gases and are thus a crucial component of the global climate system [2, 3]. Despite harsh conditions, tundra soils harbour highly diverse and specialized micro-organisms, most of which still remain unknown [4–8]. Fuelled by ever-increasing sequencing and computational power, recent culture-independent metagenome investigations have revealed that uncultured micro-organisms – the so-called ‘microbial dark matter’ – play critical roles in the carbon [4–6] and nitrogen [6–8] cycles in the tundra. More studies on the diversity and functional capacity of tundra microbiomes are thus needed to understand better their contribution to global biogeochemical cycles and the GHG budget. Here, we report 59 metagenome-assembled genomes (MAGs) recovered from tundra soils in northern Norway.

## Description of the dataset

We sampled soils across ten sites in an area of alpine tundra in the Rásttigáisá Fell (69 °59’ N, 26 °15′ E, 700 m.a.s.l), Finnmark, Norway. In each site, one sample was taken from the mineral layer (10–15 cm depth) with a soil corer and transferred immediately to dry ice. Soil water content ranged from 9.6–17.3 %, organic matter from 1.2–7.6 % and pH from 4.5 to 5.3. Metagenomic DNA was extracted with the DNeasy PowerSoil kit (QIAGEN, Hilden, Germany) and libraries were prepared with the Nextera XT kit (Illumina, San Diego, CA, USA) following the manufacturers’ instructions. Paired-end sequencing was done with the Illumina NextSeq500 platform (forward reads: 170 bp, reverse reads: 140 bp) at the Institute of Biotechnology, University of Helsinki. A total of 114 950 383 paired-end reads was obtained (35.6 Gb), ranging from 7 696 757 to 14 621 365 reads per sample (2.4–4.5 Gb).

Sequencing data was processed using a genome-resolved metagenomics approach. First, *Cutadapt* v1.10 [9] was used to remove adapters and base calls with Phred score <28, and the quality of the data was checked with *FastQC* v0.11.5 [10]. Sequences were then assembled into larger contiguous sequences with *MEGAHIT* v1.1.1 [11]. Assemblies were done for each sample individually and as one co-assembly consisting of pooled data from all samples. For each assembly, contigs≥2500 bp were binned with *anvi’o* v6.0 [12] according to Pessi *et al*. [8]. Briefly, MAGs were obtained by manually grouping the contigs according to tetranucleotide frequency and differential coverage using the *anvi-interactive* interface of *anvi’o*. MAGs were visually inspected to remove outlying contigs, and only MAGs with homogeneous sequence composition and coverage were kept (see Fig. S1 and merenlab.org/2015/05/11/anvi-refine for more information). MAGs from all assemblies were combined and *dRep* v2.3.2 [13] was used to yield a set of 59 non-redundant MAGs (≥99 % average nucleotide identity).

**Fig. 1.**
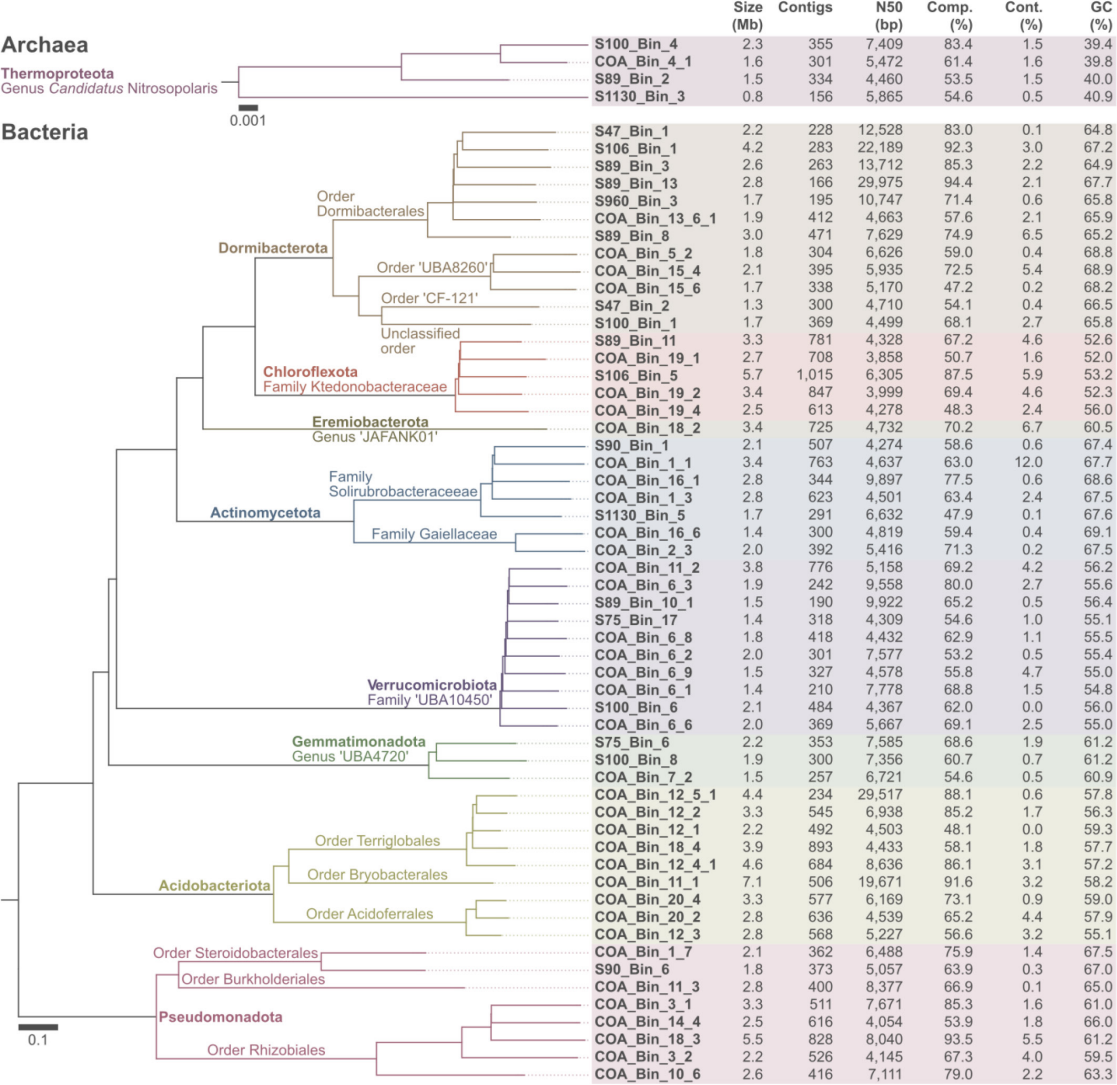
Fifty-nine metagenome-assembled genomes (MAGs) from mineral tundra soils in Rásttigáisá, northern Norway. Maximum-likelihood trees obtained with *GTDB-Tk* v2.3.0 based on 53 archaeal and 120 bacterial single-copy genes. Completion and redundancy values were obtained with *Checkm2* v1.0.1.

The 59 non-redundant MAGs range from 0.8 to 7.1 Mb and comprise 156–1015 contigs with N_50_ values of 3858–29 975 bp (Fig. 1, Table S1). Completeness and contamination levels estimated with *CheckM2* v1.0.1 [14] are of 47.2–94.4 % and 0.0–12.0 %, respectively. These estimates are based on machine-learning models trained on simulated genomes, which seem to give more reliable estimates than previous single-copy gene-based approaches [14]. GC content ranges from 40.0–69.0 %. Taxonomic classification with *GTDB-Tk* v2.3.0 [15] and the Genome Taxonomy Database (GTDB) release R214 [16] placed the MAGs within the bacterial phyla *Candidatus* Dormibacterota (*n*=12), Verrucomicrobiota (*n*=10), Acidobacteriota (*n*=9), Pseudomonadota (*n*=8), Actinomycetota (*n*=7), Chloroflexota (*n*=5), Gemmatimonadota (*n*=3) and Eremiobacterota (*n*=1). The remaining MAGs (*n*=4) were assigned to the archaeal phylum Thermoproteota. These have been characterized elsewhere as part of the recently proposed genus *Candidatus* Nitrosopolaris [8].

## Future outlook

The 59 Rásttigáisá MAGs obtained here represent medium- and high-quality genomes from microbial populations in mineral tundra soils. Among other applications, these population genomes can be used in studies of microbial phylogenomics, biogeography and comparative genomics looking at adaptations to life under cold, oligotrophic conditions. An example of this is the study by Pessi *et al*. [8], in which four of the MAGs reported here were included in the description of a novel genus of putative ammonia-oxidizing archaea that appears to be restricted to polar and alpine environments. Our preliminary analysis of the Rásttigáisá MAGs suggests that other novel taxa might be represented in this dataset (Table S1), but further studies are needed for a better characterization of this potentially novel diversity. The prevalence of uncultured taxa has also been observed in other metagenomic investigations of tundra soils in Alaska [4, 5], Finland [6, 7], Sweden [17], as well as Antarctic soils [18, 19]. In particular, analysis of the MAGs assigned to *Candidatus* Dormibacterota, which is the most abundant phylum in the dataset, can cast light into the ecological roles of this quite enigmatic group of micro-organisms in cold and oligotrophic soils [18, 19]. Further functional annotation and metabolic reconstruction of the Rásttigáisá MAGs can provide clues about the potential roles of these populations in the cycling of GHGs [7, 17]. Despite the small scale when considered individually, this and other similar studies [20–22] contribute to increasing the coverage of the polar microbiome census.

## Supplementary Data

Supplementary material 1
