# Supplementary material for: Metagenome-assembled genomes from mineral tundra soils in Rásttigáisá, northern Norway
Source: Access Microbiol. 2024 Jan 23;6(1):000655.v3. doi: 10.1099/acmi.0.000655.v3 (PMC10866031; doi:10.1099/acmi.0.000655.v3)
Supplement: Supplementary material 1 [file acmi-6-655.v3-s001.pdf]

**Table S1.** Fifty-nine metagenome-assembled genomes (MAGs) from mineral tundra soils in Rásttigáisá, northern Norway.

| MAG | ENA accession | CheckM2 v1.0.1 | | | | | | |
|---|---|---|---|---|---|---|---|---|
| | | Completeness | Contamination | Contig $N_{50}$ | Genome size | GC content | Coding sequences | Coding density |
| COA_Bin_4_1 | GCA_933227015.1 | 61.4 | 1.6 | 5,472 | 1,550,055 | 40.0 | 2,024 | 0.8 |
| S100_Bin_4 | GCA_933226995.1 | 83.4 | 1.5 | 7,409 | 2,260,634 | 40.0 | 2,851 | 0.7 |
| S89_Bin_2 | GCA_933227005.1 | 53.5 | 1.5 | 4,460 | 1,485,530 | 40.0 | 1,952 | 0.8 |
| S1130_Bin_3 | GCA_933226985.1 | 54.6 | 0.5 | 5,865 | 810,077 | 41.0 | 931 | 0.8 |
| COA_Bin_12_3 | GCA_955652425.1 | 56.6 | 3.2 | 5,227 | 2,797,217 | 55.0 | 2,882 | 0.9 |
| COA_Bin_20_2 | GCA_955652085.1 | 65.2 | 4.4 | 4,539 | 2,803,403 | 58.0 | 2,985 | 0.9 |
| COA_Bin_20_4 | GCA_955651735.1 | 73.1 | 0.9 | 6,169 | 3,259,331 | 59.0 | 3,331 | 0.9 |
| COA_Bin_11_1 | GCA_955652915.1 | 91.6 | 3.2 | 19,671 | 7,085,949 | 58.0 | 6,397 | 0.9 |
| COA_Bin_12_2 | GCA_955652845.1 | 85.2 | 1.7 | 6,938 | 3,290,052 | 56.0 | 3,318 | 0.9 |
| COA_Bin_12_1 | GCA_955652275.1 | 48.1 | 0.0 | 4,503 | 2,180,510 | 59.0 | 2,310 | 0.9 |
| COA_Bin_12_5_1 | GCA_955652395.1 | 88.1 | 0.6 | 29,517 | 4,420,380 | 58.0 | 4,299 | 0.8 |
| COA_Bin_18_4 | GCA_955651745.1 | 58.1 | 1.8 | 4,433 | 3,858,378 | 58.0 | 4,355 | 0.8 |
| COA_Bin_12_4_1 | GCA_955652315.1 | 86.1 | 3.1 | 8,636 | 4,635,975 | 57.0 | 4,644 | 0.9 |
| COA_Bin_16_6 | GCA_955652375.1 | 59.4 | 0.4 | 4,819 | 1,395,090 | 69.0 | 1,680 | 0.9 |
| COA_Bin_2_3 | GCA_955652075.1 | 71.3 | 0.2 | 5,416 | 2,008,063 | 67.0 | 2,447 | 0.9 |
| COA_Bin_1_1 | GCA_955652575.1 | 63.0 | 12.0 | 4,637 | 3,443,195 | 68.0 | 4,100 | 0.9 |
| COA_Bin_1_3 | GCA_955652295.1 | 63.4 | 2.4 | 4,501 | 2,758,223 | 68.0 | 3,285 | 0.9 |
| COA_Bin_16_1 | GCA_955652345.1 | 77.5 | 0.6 | 9,897 | 2,753,912 | 69.0 | 3,007 | 0.9 |
| S1130_Bin_5 | GCA_955652215.1 | 47.9 | 0.1 | 6,632 | 1,679,047 | 68.0 | 1,918 | 0.9 |
| S90_Bin_1 | GCA_955652515.1 | 58.6 | 0.6 | 4,274 | 2,102,392 | 67.0 | 2,461 | 0.9 |
| COA_Bin_19_2 | GCA_955652695.1 | 69.4 | 4.6 | 3,999 | 3,389,269 | 52.0 | 3,492 | 0.9 |
| S89_Bin_11 | GCA_955652885.1 | 67.2 | 4.6 | 4,328 | 3,299,544 | 52.0 | 3,324 | 0.9 |
| COA_Bin_19_4 | GCA_955652175.1 | 48.3 | 2.4 | 4,278 | 2,539,617 | 56.0 | 2,632 | 0.9 |
| COA_Bin_19_1 | GCA_955652765.1 | 50.7 | 1.6 | 3,858 | 2,743,106 | 52.0 | 2,769 | 0.9 |
| S106_Bin_5 | GCA_955652655.1 | 87.5 | 5.9 | 6,305 | 5,667,277 | 53.0 | 5,839 | 0.9 |
| S47_Bin_2 | GCA_955652415.1 | 54.1 | 0.4 | 4,710 | 1,344,415 | 66.0 | 1,551 | 0.9 |
| S100_Bin_1 | GCA_955652475.1 | 68.1 | 2.7 | 4,499 | 1,666,936 | 66.0 | 2,003 | 0.9 |
| S47_Bin_1 | GCA_955652565.1 | 83.0 | 0.1 | 12,528 | 2,223,009 | 65.0 | 2,406 | 0.9 |
| S89_Bin_3 | GCA_955652835.1 | 85.3 | 2.2 | 13,712 | 2,627,168 | 65.0 | 2,896 | 0.9 |
| S106_Bin_1 | GCA_955652675.1 | 92.3 | 3.0 | 22,189 | 4,211,831 | 68.0 | 4,389 | 0.9 |
| COA_Bin_13_6_1 | GCA_955652285.1 | 57.6 | 2.1 | 4,663 | 1,867,287 | 66.0 | 2,281 | 0.9 |
| S89_Bin_8 | GCA_955652535.1 | 74.9 | 6.5 | 7,629 | 3,005,496 | 65.0 | 3,442 | 0.9 |
| S960_Bin_3 | GCA_955652585.1 | 71.4 | 0.6 | 10,747 | 1,689,212 | 66.0 | 1,851 | 0.9 |
| S89_Bin_13 | GCA_955652705.1 | 94.4 | 2.1 | 29,975 | 2,792,386 | 68.0 | 2,978 | 0.9 |
| COA_Bin_15_4 | GCA_955652545.1 | 72.5 | 5.4 | 5,935 | 2,113,622 | 69.0 | 2,416 | 0.9 |
| COA_Bin_15_6 | GCA_955652455.1 | 47.2 | 0.2 | 5,170 | 1,682,440 | 68.0 | 1,936 | 0.9 |
| COA_Bin_5_2 | GCA_955651875.1 | 59.0 | 0.4 | 6,626 | 1,837,968 | 69.0 | 2,061 | 0.9 |
| COA_Bin_18_2 | GCA_955652605.1 | 70.2 | 6.7 | 4,732 | 3,373,578 | 60.0 | 3,808 | 0.9 |
| COA_Bin_7_2 | GCA_955652265.1 | 54.6 | 0.5 | 6,721 | 1,518,907 | 61.0 | 1,617 | 0.9 |
| S100_Bin_8 | GCA_955652665.1 | 60.7 | 0.7 | 7,356 | 1,899,585 | 61.0 | 2,029 | 0.9 |
| S75_Bin_6 | GCA_955652635.1 | 68.6 | 1.9 | 7,585 | 2,215,377 | 61.0 | 2,347 | 0.9 |
| COA_Bin_3_2 | GCA_955651925.1 | 67.3 | 4.0 | 4,145 | 2,172,092 | 60.0 | 2,508 | 0.8 |
| COA_Bin_10_6 | GCA_955652235.1 | 79.0 | 2.2 | 7,111 | 2,583,449 | 64.0 | 2,879 | 0.9 |
| COA_Bin_14_4 | GCA_955652205.1 | 53.9 | 1.8 | 4,054 | 2,489,135 | 66.0 | 2,784 | 0.9 |
| COA_Bin_3_1 | GCA_955652195.1 | 85.3 | 1.6 | 7,671 | 3,300,738 | 61.0 | 3,594 | 0.9 |
| COA_Bin_18_3 | GCA_955652925.1 | 93.5 | 5.5 | 8,040 | 5,539,610 | 61.0 | 5,756 | 0.9 |
| COA_Bin_11_3 | GCA_955652025.1 | 66.9 | 0.1 | 8,377 | 2,775,195 | 65.0 | 2,959 | 0.9 |
| COA_Bin_1_7 | GCA_955652795.1 | 75.9 | 1.4 | 6,488 | 2,093,306 | 68.0 | 2,201 | 0.9 |
| S90_Bin_6 | GCA_955652625.1 | 63.9 | 0.3 | 5,057 | 1,769,326 | 67.0 | 1,914 | 0.9 |
| COA_Bin_11_2 | GCA_955652855.1 | 69.2 | 4.2 | 5,158 | 3,791,130 | 56.0 | 4,393 | 0.9 |
| COA_Bin_6_8 | GCA_955652895.1 | 62.9 | 1.1 | 4,432 | 1,779,237 | 55.0 | 2,018 | 0.9 |
| S100_Bin_6 | GCA_955652735.1 | 62.0 | 0.0 | 4,367 | 2,063,516 | 56.0 | 2,386 | 0.9 |
| S89_Bin_10_1 | GCA_955652185.1 | 65.2 | 0.5 | 9,922 | 1,469,019 | 56.0 | 1,521 | 0.9 |
| COA_Bin_6_2 | GCA_955652155.1 | 53.2 | 0.5 | 7,577 | 1,956,077 | 55.0 | 2,163 | 0.9 |
| COA_Bin_6_3 | GCA_955652255.1 | 80.0 | 2.7 | 9,558 | 1,889,877 | 56.0 | 2,085 | 0.9 |
| COA_Bin_6_1 | GCA_955652245.1 | 68.8 | 1.5 | 7,778 | 1,384,978 | 55.0 | 1,542 | 0.9 |
| COA_Bin_6_6 | GCA_955652715.1 | 69.1 | 2.5 | 5,667 | 1,984,330 | 55.0 | 2,296 | 0.9 |
| COA_Bin_6_9 | GCA_955652555.1 | 55.8 | 4.7 | 4,578 | 1,458,125 | 55.0 | 1,694 | 0.9 |
| S75_Bin_17 | GCA_955652225.1 | 54.6 | 1.0 | 4,309 | 1,363,746 | 55.0 | 1,616 | 0.9 |

**Table S1.** Continued.

| MAG | GTDB-Tk v2.3.0 (GTDB R214) | | |
|---|---|---|---|
| | Classification | MSA percent | Red value |
| COA_Bin_4_1 | d__Archaea;p__Thermoproteota;c__Nitrososphaeria;o__Nitrososphaerales;f__Nitrososphaeraceae;g__Nitrosopolaris;s__Nitrosopolaris rasttigaisensis | 56.3 | - |
| S100_Bin_4 | d__Archaea;p__Thermoproteota;c__Nitrososphaeria;o__Nitrososphaerales;f__Nitrososphaeraceae;g__Nitrosopolaris;s__Nitrosopolaris rasttigaisensis | 75.9 | - |
| S89_Bin_2 | d__Archaea;p__Thermoproteota;c__Nitrososphaeria;o__Nitrososphaerales;f__Nitrososphaeraceae;g__Nitrosopolaris;s__Nitrosopolaris rasttigaisensis | 47.0 | - |
| S1130_Bin_3 | d__Archaea;p__Thermoproteota;c__Nitrososphaeria;o__Nitrososphaerales;f__Nitrososphaeraceae;g__Nitrosopolaris;s__Nitrosopolaris sp009665115 | 63.9 | - |
| COA_Bin_12_3 | d__Bacteria;p__Acidobacteriota;c__Terriglobia;o__Acidoferrales;f__UBA7541;g__Acidoferrum;s__ | 55.5 | 0.9 |
| COA_Bin_20_2 | d__Bacteria;p__Acidobacteriota;c__Terriglobia;o__Acidoferrales;f__UBA7541;g__Acidoferrum;s__ | 49.6 | 0.9 |
| COA_Bin_20_4 | d__Bacteria;p__Acidobacteriota;c__Terriglobia;o__Acidoferrales;f__UBA7541;g__Acidoferrum;s__ | 65.6 | 0.9 |
| COA_Bin_11_1 | d__Bacteria;p__Acidobacteriota;c__Terriglobia;o__Bryobacterales;f__Bryobacteraceae;g__Palsa-89;s__ | 85.1 | 0.9 |
| COA_Bin_12_2 | d__Bacteria;p__Acidobacteriota;c__Terriglobia;o__Terriglobales;f__SbA1;g__JABCYV01;s__ | 79.3 | 1.0 |
| COA_Bin_12_1 | d__Bacteria;p__Acidobacteriota;c__Terriglobia;o__Terriglobales;f__SbA1;g__JAIQFF01;s__ | 45.5 | 1.0 |
| COA_Bin_12_5_1 | d__Bacteria;p__Acidobacteriota;c__Terriglobia;o__Terriglobales;f__SbA1;g__JAIQFF01;s__ | 68.6 | 1.0 |
| COA_Bin_18_4 | d__Bacteria;p__Acidobacteriota;c__Terriglobia;o__Terriglobales;f__SbA1;g__JAIQFF01;s__ | 46.2 | 1.0 |
| COA_Bin_12_4_1 | d__Bacteria;p__Acidobacteriota;c__Terriglobia;o__Terriglobales;f__SbA1;g__Sulfotelmatobacter;s__ | 52.1 | 1.0 |
| COA_Bin_16_6 | d__Bacteria;p__Actinomycetota;c__Thermoleophilia;o__Gaiellales;f__Gaiellaceae;g__13-2-20CM-68-14;s__ | 52.2 | 0.9 |
| COA_Bin_2_3 | d__Bacteria;p__Actinomycetota;c__Thermoleophilia;o__Gaiellales;f__Gaiellaceae;g__3-1-20CM-4-69-9;s__ | 64.7 | 1.0 |
| COA_Bin_1_1 | d__Bacteria;p__Actinomycetota;c__Thermoleophilia;o__Solirubrobacterales;f__Solirubrobacteraceae;g__Palsa-465;s__ | 40.3 | 0.9 |
| COA_Bin_1_3 | d__Bacteria;p__Actinomycetota;c__Thermoleophilia;o__Solirubrobacterales;f__Solirubrobacteraceae;g__Palsa-465;s__ | 40.3 | 0.9 |
| COA_Bin_16_1 | d__Bacteria;p__Actinomycetota;c__Thermoleophilia;o__Solirubrobacterales;f__Solirubrobacteraceae;g__Palsa-465;s__ | 52.9 | 0.9 |
| S1130_Bin_5 | d__Bacteria;p__Actinomycetota;c__Thermoleophilia;o__Solirubrobacterales;f__Solirubrobacteraceae;g__Palsa-465;s__ | 37.8 | 0.9 |
| S90_Bin_1 | d__Bacteria;p__Actinomycetota;c__Thermoleophilia;o__Solirubrobacterales;f__Solirubrobacteraceae;g__Palsa-465;s__ | 48.5 | 0.9 |
| COA_Bin_19_2 | d__Bacteria;p__Chloroflexota;c__Ktedonobacteria;o__Ktedonobacterales;f__Ktedonobacteraceae;g__;s__ | 65.7 | 0.9 |
| S89_Bin_11 | d__Bacteria;p__Chloroflexota;c__Ktedonobacteria;o__Ktedonobacterales;f__Ktedonobacteraceae;g__;s__ | 68.2 | 0.9 |
| COA_Bin_19_4 | d__Bacteria;p__Chloroflexota;c__Ktedonobacteria;o__Ktedonobacterales;f__Ktedonobacteraceae;g__CF-113;s__ | 53.7 | 0.9 |
| COA_Bin_19_1 | d__Bacteria;p__Chloroflexota;c__Ktedonobacteria;o__Ktedonobacterales;f__Ktedonobacteraceae;g__UBA11361;s__ | 52.7 | 0.9 |
| S106_Bin_5 | d__Bacteria;p__Chloroflexota;c__Ktedonobacteria;o__Ktedonobacterales;f__Ktedonobacteraceae;g__UBA11361;s__ | 77.8 | 1.0 |
| S47_Bin_2 | d__Bacteria;p__Dormibacterota;c__Dormibacteria;o__;f__;g__;s__ | 53.8 | 0.5 |
| S100_Bin_1 | d__Bacteria;p__Dormibacterota;c__Dormibacteria;o__CF-121;f__CF-121;g__CF-13;s__ | 62.7 | 1.0 |
| S47_Bin_1 | d__Bacteria;p__Dormibacterota;c__Dormibacteria;o__Dormibacterales;f__Dormibacteraceae;g__Palsa-870;s__ | 56.2 | 0.9 |
| S89_Bin_3 | d__Bacteria;p__Dormibacterota;c__Dormibacteria;o__Dormibacterales;f__Dormibacteraceae;g__Palsa-870;s__ | 82.8 | 0.9 |
| S106_Bin_1 | d__Bacteria;p__Dormibacterota;c__Dormibacteria;o__Dormibacterales;f__Dormibacteraceae;g__QHBS01;s__ | 87.9 | 0.9 |
| COA_Bin_13_6_1 | d__Bacteria;p__Dormibacterota;c__Dormibacteria;o__Dormibacterales;f__Dormibacteraceae;g__UBA10449;s__ | 41.0 | 1.0 |
| S89_Bin_8 | d__Bacteria;p__Dormibacterota;c__Dormibacteria;o__Dormibacterales;f__Dormibacteraceae;g__UBA10449;s__ | 53.2 | 1.0 |
| S960_Bin_3 | d__Bacteria;p__Dormibacterota;c__Dormibacteria;o__Dormibacterales;f__Dormibacteraceae;g__UBA10449;s__ | 62.8 | 1.0 |
| S89_Bin_13 | d__Bacteria;p__Dormibacterota;c__Dormibacteria;o__Dormibacterales;f__Dormibacteraceae;g__VBGR01;s__ | 89.7 | 1.0 |
| COA_Bin_15_4 | d__Bacteria;p__Dormibacterota;c__Dormibacteria;o__UBA8260;f__UBA8260;g__Palsa-875;s__ | 76.8 | 0.9 |
| COA_Bin_15_6 | d__Bacteria;p__Dormibacterota;c__Dormibacteria;o__UBA8260;f__UBA8260;g__Palsa-875;s__ | 42.1 | 0.9 |
| COA_Bin_5_2 | d__Bacteria;p__Dormibacterota;c__Dormibacteria;o__UBA8260;f__UBA8260;g__Palsa-875;s__ | 50.7 | 1.0 |
| COA_Bin_18_2 | d__Bacteria;p__Eremiobacterota;c__Eremiobacteria;o__Eremiobacterales;f__Eremiobacteraceae;g__JAFANK01;s__ | 55.5 | 0.9 |
| COA_Bin_7_2 | d__Bacteria;p__Gemmatimonadota;c__Gemmatimonadetes;o__Gemmatimonadales;f__Gemmatimonadaceae;g__UBA4720;s__ | 40.8 | 1.0 |
| S100_Bin_8 | d__Bacteria;p__Gemmatimonadota;c__Gemmatimonadetes;o__Gemmatimonadales;f__Gemmatimonadaceae;g__UBA4720;s__ | 50.9 | 1.0 |
| S75_Bin_6 | d__Bacteria;p__Gemmatimonadota;c__Gemmatimonadetes;o__Gemmatimonadales;f__Gemmatimonadaceae;g__UBA4720;s__ | 59.0 | 1.0 |
| COA_Bin_3_2 | d__Bacteria;p__Pseudomonadota;c__Alphaproteobacteria;o__Rhizobiales;f__Beijerinckiaceae;g__Methylocella;s__Methylocella sp006514895 | 61.2 | - |
| COA_Bin_10_6 | d__Bacteria;p__Pseudomonadota;c__Alphaproteobacteria;o__Rhizobiales;f__Methyloligellaceae;g__Methyloceanibacter;s__Methyloceanibacter sp002383105 | 72.2 | - |
| COA_Bin_14_4 | d__Bacteria;p__Pseudomonadota;c__Alphaproteobacteria;o__Rhizobiales;f__Xanthobacteraceae;g__AP-16;s__ | 51.8 | 1.0 |
| COA_Bin_3_1 | d__Bacteria;p__Pseudomonadota;c__Alphaproteobacteria;o__Rhizobiales;f__Xanthobacteraceae;g__BOG-931;s__ | 79.1 | 1.0 |
| COA_Bin_18_3 | d__Bacteria;p__Pseudomonadota;c__Alphaproteobacteria;o__Rhizobiales;f__Xanthobacteraceae;g__VAZQ01;s__ | 83.5 | 0.9 |
| COA_Bin_11_3 | d__Bacteria;p__Pseudomonadota;c__Gammaproteobacteria;o__Burkholderiales;f__Casimicrobiaceae;g__VBCG01;s__ | 52.6 | 0.9 |
| COA_Bin_1_7 | d__Bacteria;p__Pseudomonadota;c__Gammaproteobacteria;o__Steroidobacterales;f__Steroidobacteraceae;g__13-2-20CM-66-19;s__ | 70.6 | 1.0 |
| S90_Bin_6 | d__Bacteria;p__Pseudomonadota;c__Gammaproteobacteria;o__Steroidobacterales;f__Steroidobacteraceae;g__13-2-20CM-66-19;s__ | 52.1 | 1.0 |
| COA_Bin_11_2 | d__Bacteria;p__Verrucomicrobiota;c__Verrucomicrobiae;o__Chthoniobacterales;f__UBA10450;g__;s__ | 57.5 | 0.9 |
| COA_Bin_6_8 | d__Bacteria;p__Verrucomicrobiota;c__Verrucomicrobiae;o__Chthoniobacterales;f__UBA10450;g__;s__ | 49.9 | 0.9 |
| S100_Bin_6 | d__Bacteria;p__Verrucomicrobiota;c__Verrucomicrobiae;o__Chthoniobacterales;f__UBA10450;g__AV133;s__ | 52.5 | 0.9 |
| S89_Bin_10_1 | d__Bacteria;p__Verrucomicrobiota;c__Verrucomicrobiae;o__Chthoniobacterales;f__UBA10450;g__AV133;s__AV133 sp003219265 | 53.1 | - |
| COA_Bin_6_2 | d__Bacteria;p__Verrucomicrobiota;c__Verrucomicrobiae;o__Chthoniobacterales;f__UBA10450;g__AV40;s__ | 38.2 | 1.0 |
| COA_Bin_6_3 | d__Bacteria;p__Verrucomicrobiota;c__Verrucomicrobiae;o__Chthoniobacterales;f__UBA10450;g__UBA10450;s__ | 74.2 | 1.0 |
| COA_Bin_6_1 | d__Bacteria;p__Verrucomicrobiota;c__Verrucomicrobiae;o__Chthoniobacterales;f__UBA10450;g__Udaeobacter;s__ | 55.0 | 1.0 |
| COA_Bin_6_6 | d__Bacteria;p__Verrucomicrobiota;c__Verrucomicrobiae;o__Chthoniobacterales;f__UBA10450;g__Udaeobacter;s__ | 50.7 | 1.0 |
| COA_Bin_6_9 | d__Bacteria;p__Verrucomicrobiota;c__Verrucomicrobiae;o__Chthoniobacterales;f__UBA10450;g__Udaeobacter;s__ | 45.3 | 1.0 |
| S75_Bin_17 | d__Bacteria;p__Verrucomicrobiota;c__Verrucomicrobiae;o__Chthoniobacterales;f__UBA10450;g__Udaeobacter;s__ | 42.6 | 0.9 |

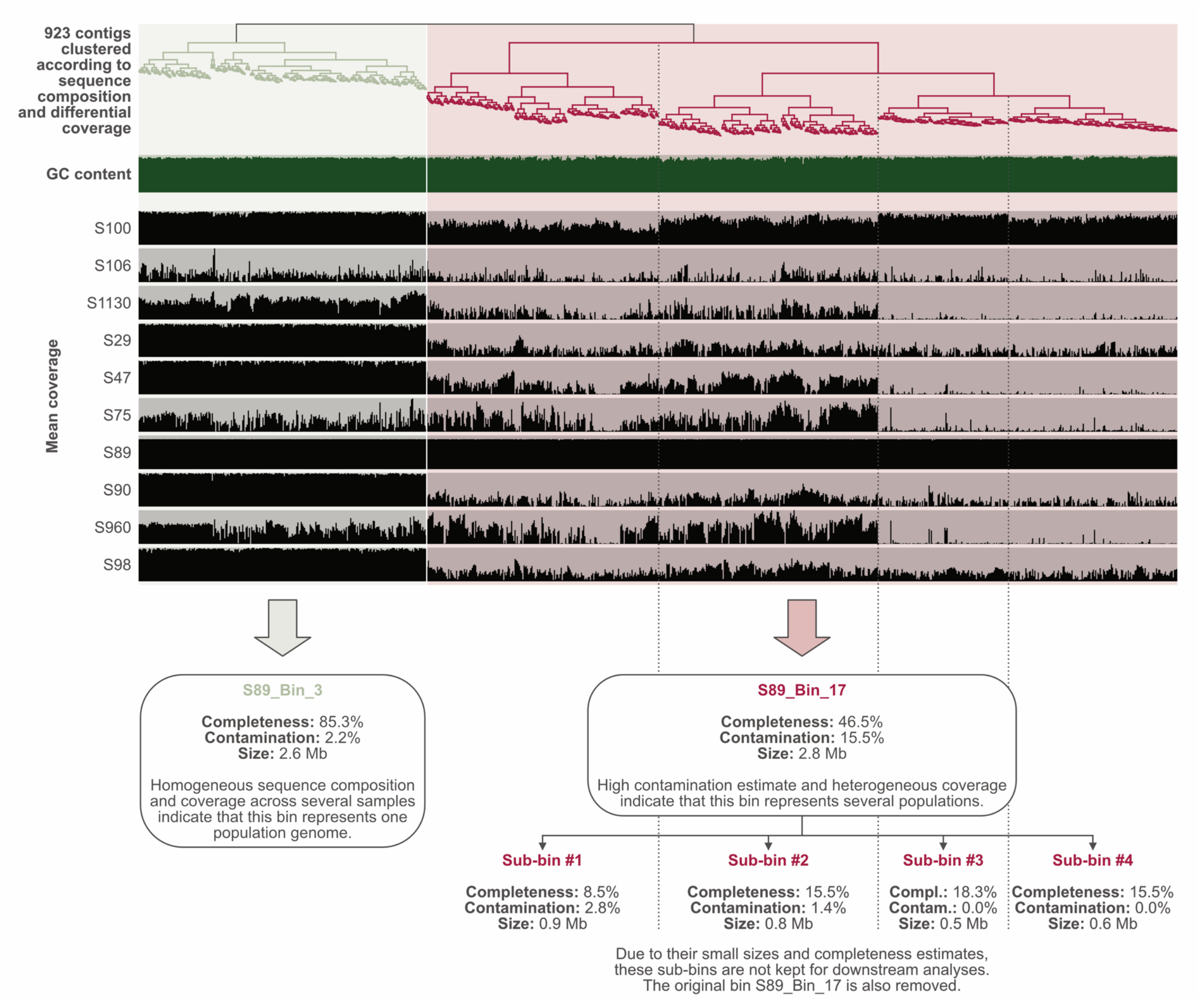


**Figure S1.** Diagram showing examples of a good- and a bad-quality metagenomic bin. Made with *anvi'o* v6.0 (Eren *et al*., 2021).
